# When Definitions Differ, are Comparisons Meaningful? Definitions of Weight Regain After Bariatric Surgery and Their Associations with Patient Characteristics and Clinical Outcomes - A Need for a Revisit?

**DOI:** 10.1007/s11695-023-06528-z

**Published:** 2023-03-30

**Authors:** Wahiba Elhag, Merilyn Lock, Walid El Ansari

**Affiliations:** 1 Department of Bariatric Surgery/Bariatric Medicine, Hamad Medical Corporation, Doha, Qatar; 2 Weill Cornell Medicine-Qatar, Doha, Qatar; 3 Division of Exercise Science, Health and Epidemiology, College of Health and Life Sciences, Hamad Bin Khalifa University, Doha, Qatar; 4 Department of Surgery, Hamad Medical Corporation, Doha, Qatar; 5 College of Medicine, Qatar University, Doha, Qatar

**Keywords:** Bariatric surgery, Laparoscopic sleeve gastrectomy, Definition, Weight regain, Predictors of weight regain, Remission

## Abstract

**Introduction:**

Definitions and prevalence of weight regain (WR) after bariatric surgery remains inconsistent and their clinical significance unclear.

**Objectives:**

To assess WR five years after sleeve gastrectomy (LSG), employing six definitions; and appraise their association with patient characteristics/clinical outcomes.

**Methods:**

Consecutive patients (*N* = 589) who underwent LSG were followed up for 5 years. WR prevalence was calculated yearly employing six definitions. Regression analysis assessed associations between WR at 5 years, and patient characteristics (age, sex, preop BMI, number of follow-up visits, number of comorbidities) and remission of comorbidities (type 2 diabetes, hypertension, and dyslipidemia).

**Results:**

Sample’s mean age and BMI were 34 ± 11.6 years and 43.13 ± 5.77 kg/m^2^, and 64% were females. Percentage of patients with WR at 2, 3, 4, and 5 years fluctuated between 2.53% and 94.18%, subject to definition, and time point. The definition “Any WR” generated the highest prevalence of WR (86–94%) across all time points. At 5 years, for patient characteristics, preoperative BMI was associated with three definitions (P 0.49 to < 0.001), sex was associated with two (*P* < 0.026–0.032), and number of comorbidities was associated with one definition (*P* = 0.01). In terms of comorbidities, only hypertension was associated with WR (one definition, *P* = 0.025). No other definitions of WR were associated with any of the variables under examination.

**Conclusion:**

Weight regain is reasonably expected after BMS. WR definitions were of minor clinical significance due to weak associations with limited comorbidities. Dichotomous definitions might offer some guidance while managing individual patients. However, its utility as a comparator metric across patients/procedures requires refinements.

**Graphical abstract:**

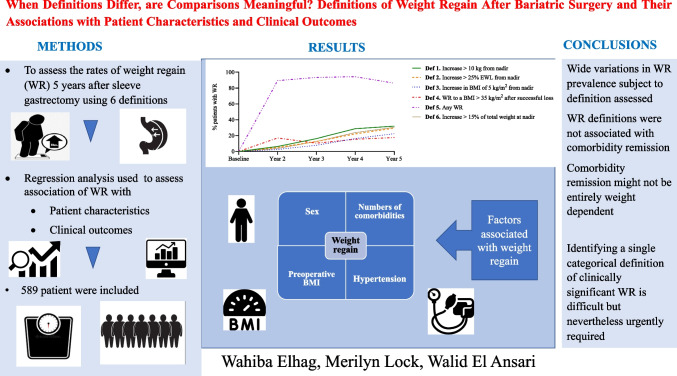

## Introduction

Due to the excellent short- and long-term weight loss (WL) and durable improvements in obesity-associated comorbidities, bariatric and metabolic surgery (BMS) is considered the best treatment for extreme obesity [1–6]. However, not all patients maintain their achieved WL after BMS, and some experience weight regain (WR). For instance, the prevalence of WR ranged between 5.7 and 76% at 2–6 years after laparoscopic sleeve gastrectomy (LSG) [7, 8]; and a meta-analysis of patients with ≥ 7 years follow-up demonstrated a long-term WR rate of 27.8% (range 14–37%) [9].

Such wide variability in the proportion of patients who regain weight after BMS probably reflects the inconsistencies in the way WR is assessed. While standard definitions for WL after BMS surgery exist [10], the lack of a standardized WR definition results in variations and therefore imprecise comparisons across studies, procedures, settings, patient groups, and countries. This contributes to our poor understanding of WR and its significance [11].

Indeed, the definitions of WR after BMS are numerous and employ a wide range of different parameters, e.g., BMI or excess weight loss percentage (EWL%) as well as different cutoffs [12–20]. There have been calls to rectify this deficiency but identifying a single definition of clinically significant WR is challenging [21, 22].

Despite such calls, sparse research has assessed the prevalence of WR using the different definitions, and the literature remains limited in number and scope. For instance, in terms of the BMS procedure, most studies assessed the effects of WR definitions after Roux en Y gastric bypass (RYGB) only or after other BMS procedures, but not after  laparoscopic sleeve gastrectomy (LSG), despite its popularity and different outcomes [12, 14, 21, 23–25]. Hence, the current study focused on LSG to bridge this gap. In addition, some studies appraised the effects of various definitions on WR rates at single time point, e.g., at five years [12, 21] without providing the prevalence of WR at multiple successive time points. While such an approach is useful, it provides a limited “single snapshot” view rather than the ongoing mechanics of the WR definitions across time. Equally important is that very few studies have evaluated how various WR definitions are associated with different comorbidities. Such understanding is critical to appraise the clinical implications of each of these definitions [14, 23]. WR has important health consequences including relapse of obesity-associated comorbidities including type 2 diabetes (T2DM), hypertension, and dyslipidemia [12, 24–28]

Given the above knowledge gaps, the aim of the present study was to assess the prevalence of WR after primary LSG using the different definitions. The specific objectives were to evaluate the extent of variability of WR rates based on six definitions measured at four time points (years 2, 3, 4, and 5 after LSG); and, appraise the associations between the different WR definitions and a range of patient characteristics as well as the remission of three comorbidities at 5 years after LSG. Clearer definitions will help to understand the clinical significance of such definitions, and hence provide guidance when intervention is required [11, 29]. We selected LSG as it is the most common procedure performed at our institution, comprising almost 95% of our surgeries. Patients with revisional BMS were excluded as nadir weight, which is essential for four of the six WR definitions we examined, would be difficult to determine precisely [14].

## Materials and Methods

### Study Design, Ethics, and Participants

This retrospective study was approved by the Medical Research Centre at Hamad Medical Corporation in Doha, Qatar (IRB Protocol #MRC-01–20-658). The inclusion criteria were all adults aged ≥ 18 years with BMI ≥ 40 or BMI ≥ 35 kg/m^2^ with comorbidities who underwent primary LSG at the BMS Centre in our institution and had ≥ 5 years follow-up data. Hence, patients operated upon from February 2016 to September of 2017 were eligible. A total of 598 patients underwent LSG during this time period. Eleven of these subsequently underwent revisional surgery and were excluded. The remaining 587 patients were included in the current analysis. The follow-up rate was 88.5, 85.8, 81, 74.9, and 61.1% at years 1, 2, 3, 4, and 5 respectively. The BMS service at our institution includes pre- and postoperative care provided in line with standard international guidelines [30]. The surgical technique of LSG at our institution has been described elsewhere and has not changed much since the establishment of the BMS department [5, 31].

### Procedures and Data Collection

We searched patients’ electronic records and retrieved pre- as well postoperative data across the five years. Information retrieved included demographics (age, sex) anthropometric measures (weight, height) data, and other patient characteristics [(number of follow-up visits, number of comorbidities, medication use (based on pharmacy dispense records and physician documentation)]. We also retrieved clinical data [systolic and diastolic blood pressure (SBP, DBP)], as well as cardiometabolic/laboratory values [total cholesterol (TC), triglycerides (TG), high-density lipoprotein (HDL), low-density lipoprotein (LDL)].

#### Weight Loss and Regain Measures

We computed the BMI, BMI change, excess weight (EW), excess weight loss percentage (EWL%), weight loss (WL), and total weight loss percentage (TWL%) at 1, 2, 3, 4, and 5 years after surgery using formulas recommended in previous studies [10, 31, 32]. Nadir weight was the lowest value achieved of all postoperative weight measures available prior to year five. WR was calculated at 4 time points (starting from the second year) based on the six definitions. Definitions, cutoffs, and calculations of WR are shown in Table 1.

**Table 1:** Six definitions of weight regain

Definition	Calculation
Def 1. Increase > 10 kg from nadir	(Total body weight in kg at FU point − total body weight in kg at nadir) > 10 kg
Def 2. Increase > 25% EWL from nadir	(EWL at nadir − EWL at FU) point > 25
Def 3. Increase in BMI of 5 kg/m^2^ from nadir	(BMI at FU point − BMI at nadir) > 5
Def 4. WR to a BMI > 35 kg/m^2^ after successful loss	BMI > 35 at FU point and successful loss to a BMI < 35. Successful loss was defined as EWL > 50% at nadir
Def 5. Any WR	(Total body weight in kg at FU point − total body weight in kg at nadir) > 0
Def 6. Increase > 15% of total weight at nadir	[(Total body weight in kg at FU point − total body weight in kg at nadir) / Total body weight at nadir in kg] × 100 > 15

Adapted from [21], *Def* definition, *WR* weight regain, *FU* follow-up, *EWL* excess weight loss,  *BMI* body mass index

#### Definitions of Comorbidities and their Remission

The presence of the three comorbidities prior to surgery were retrieved and ascertained from the patients’ electronic records including type 2 diabetes (T2DM), hypertension (HTN), and dyslipidemia. Such patients were either already diagnosed before presenting to our bariatric clinic or diagnosed when screened prior to surgery according to agreed international standards [10, 33, 34]. At 5-year follow-up, the status of these comorbidities was determined using the ASMBS guidelines [10].

#### The Bariatric and Metabolic Service: Standard Postoperative Care

The evolution and components of the Bariatric and Metabolic Surgery Department at our institution have been detailed elsewhere [35]. After surgery, patients are routinely followed by a multidisciplinary team of bariatric surgeons, physicians, dietitians and physiotherapists. Follow-up visits are scheduled at 2 weeks and then at 1, 3, 6, and 12 months, and yearly thereafter. Dieticians and physical therapists individually counsel patients on the routine post-surgery dietary intake and physical activity in accordance with international guidelines.

### Statistical Analysis

All values for weight loss and regain were calculated in Microsoft Excel using basic descriptive statistics and customized formulae. Categorical variables were expressed as frequencies and percentages, and continuous variables as means and standard deviations. Logic tests were used to determine patients who met individual definitions at each time point according to the definitions outlined in Table 1 and by others [21].

Binary logistic regression was undertaken to assess the associations between baseline patient characteristics (age, sex, preop BMI, number of follow-up visits, number of comorbidities) and WR at five years according to each of the six definitions. Logistic regression was also used to test the associations between WR by each definition with the remission of the three comorbidities under examination (hypertension, type 2 diabetes mellitus, and dyslipidemia). Assumptions regarding regression were tested and met. IBM SPSS Statistics version 20 (UK) was used for the analysis, and *P* value < 0.05 was considered statistically significant.

## Results

### Study Population: Preoperative and Selected Postoperative Characteristics

Table 2 depicts that the mean age of patients was 34 years and females were slightly more represented. More than half the sample were married and about three quarters were current smokers. Mean BMI was 43.13 kg/m^2^. The most common comorbidities were dyslipidemia, T2DM, and hypertension (about one fifth of the sample each). These were followed by prediabetes, asthma, hypothyroidism and back pain (10–11% each). Gastroesophageal reflux disease, obstructive sleep apnea, and coronary artery disease were less common (2–9% each). Other comorbidities were rare (< 1%). Mean blood pressure of the sample was normal. Mean number of follow-up visits with the bariatric clinics were 3.1 visits.

**Table 2:** Preoperative and selected postoperative characteristics of patients (*N* = 587)

Characteristic	Value
Preoperative	
Demographic	
Age at surgery (year) mean ± SD	34 ± 11.6
Married *n* (%)	303 (57.06)
Single *n* (%)	228 (42.94)
Female *n* (%)	377 (64.23)
Current smoker *n* (%)	71 (12.96)
Anthropometric mean ± SD	
Weight (kg)	115.85 ± 19.58
Height (cm)	163.76 ± 9.58
BMI (kg/m^2^)	43.12 ± 5.78
Excess weight (kg)	48.57 ± 16.07
Comorbidities *n* (%)	
Dyslipidemia	122 (20.78)
Type2 diabetes	113 (19.25)
Duration (years) mean ± SD	7.53 ± 5.87
Hypertension	110 (18.74)
Prediabetes	68 (11.64)
Asthma	65 (11.07)
Hypothyroidism	61 (10.39)
Back pain	61 (10.39)
Knee pain/OA	61 (10.39)
GERD	51 (8.69)
OSA	13 (2.21)
CAD	15 (2.56)
Fatty liver disease	11 (1.87)
CKD	4 (0.68)
DVT/PE	4 (0.68)
Depression/anxiety	4 (0.68)
Gallbladder disease	3 (0.51)
Gout	3 (0.51)
Seizure	2 (0.34)
Clinical (mean ± SD)	
SBP	129.05 ± 14.56
DBP	76.32 ± 9.86
Postoperative	
Number of follow-up visits over 5 years*	
Mean ± SD	3.12 ± 3.37

*OSA* obstructive sleep apnea, *CAD* coronary artery disease, *CKD* chronic kidney disease, *OA* osteoarthritis, *DVT/PE* deep vein thrombosis/pulmonary embolism, *GERD* Gastroesophageal reflux disease, *SBP* systolic blood pressure, *DBP* diastolic blood pressure, *visits to our multidisciplinary specialized bariatric clinics

### Anthropometric Characteristics at 5 Time Points

Across the total population, both weight and BMI showed a steady decrease until year 3 followed by a slight increase to year 5, echoed by the weight loss that displayed a steady decrease until year 3 followed by a slight increase to year 5 (Table 3). BMI change fluctuated to reach its maximum in year 2 (13.96 kg/m^2^) and its minimum in year 5 (11.67 kg/m^2^). Both %EWL and %TWL increased until year 2 and then started slightly decreasing up to year 5.

**Table 3:** Postoperative characteristics at 5 time points for the total population

Parameter	Year 1 (*n* = 520)	Year 2 (*n* = 504)	Year 3 (*n* = 476)	Year 4 (*n* = 434)	Year 5 (*n* = 359)
Weight (kg)	80.85 ± 15.12	77.57 ± 14.04	79.49 ± 14.34	81.54 ± 15.47	83.10 ± 15.68
BMI (kg/m^2^)	30.41 ± 5.48	29.20 ± 5.32	29.92 ± 5.50	30.81 ± 5.80	31.39 ± 5.98
Weight loss (kg)	34.05 ± 13.72	37.65 ± 16.12	35.94 ± 16.42	34.03 ± 16.79	31.36 ± 15.36
BMI change* (kg/m^2^)	12.67 ± 4.58	13.98 ± 5.43	13.30 ± 5.48	12.68 ± 5.76	11.69 ± 5.32
EWL%	73.04 ± 26.23	78.86 ± 29.94	74.77 ± 29.77	70.29 ± 28.75	66.92 ± 27.74
TWL%	29.30 ± 9.50	32.02 ± 10.86	30.43 ± 10.98	28.80 ± 11.28	26.92 ± 10.88

Cell values represent mean ± standard deviation. *Mean decrease, kg/m^2^, *EWL* excess weight loss, *TWL* total weight loss, *BMI* change calculated as BMI lost (represents as a positive value). All values calculated relative to baseline.

The number and percentage of patients reaching their nadir weight at each of the 4 time points were: at year 1 (189, 36.4%), year 2 (218, 43.3%), year 3 (89, 18.7%), year 4 (73, 16.82%); and for the whole sample, mean nadir weight was 75.40 ± 13.71 kg. A total of 529 (92.97%) patients achieved successful weight loss (defined as %EWL at nadir > 50), with a mean time to achieve minimum weight of 2.08 ± 1.00 years.

### %TWL at 5 Time Points by WR Definition

**Fig. 1:**
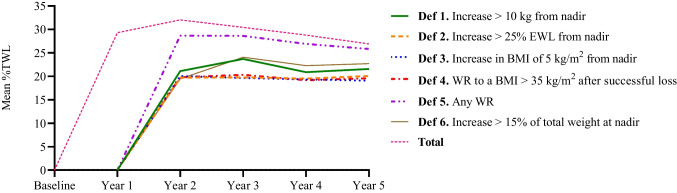
Percentage total weight loss (%TWL) over a period of 5 years for the total population and weight regain groups separately

The *%*TWL is presented in Fig. 1 for all patients and for every weight regain group separately. The %TWL for all groups was calculated relative to preoperative weight. As WR was calculated relative to nadir and the earliest time point where patients could have measured nadir was at year one, WR definitions were only applied from year 2 onwards. Definition 5 (any WR) consistently yielded the highest %TWL for any given year across all the examined years. All other definitions generated relatively lower %TWL for any given year across all the years.

### Weight Regain Rates

Table 4 depicts that during years 2, 3, 4, and 5, the percentage of patients with WR fluctuated between 2.53% and 94.18%, subject to the definition and time point. At the 5-year follow-up, the highest prevalence of WR was that of definition 5 (any WR), where about 86% of patients had experienced WR. For the remaining definitions, the percentages of patients classified as experiencing weight regain ranged between 18 and 32%.

**Table 4:** Proportion of patients with weight regain at 4 time points using different definitions

WR Definition	Year 2 (*n* = 189)	Year 3 (*n* = 407)	Year 4 (*n* = 496)	Year 5 (*n* = 569)
Def 1. Increase of > 10 kg from nadir	10 (6.33)	54 (16.27)	104 (28.81)	114 (32.02)
Def 2. Increase of > 25% EWL from nadir	8 (5.06)	42 (12.65)	80 (22.16)	105 (29.49)
Def 3. Increase in BMI of 5 kg/m^2^ from nadir	4 (2.53)	26 (7.83)	59 (16.34)	80 (22.47)
Def 4. WR to BMI > 35 kg/m^2^ after successful loss	27 (17.09)	35.00 (10.54)	56 (15.51)	63 (17.70)
Def 5. Any WR	141 (89.24)	310 (93.37)	340 (94.18)	306 (85.96)
Def 6. Increase of > 15% of TW at nadir	6 (3.80)	42 (12.65)	86 (23.82)	109 (30.62)

Cell values represent N (%), *WR* weight regain, cell values represent *N* (%), *%EWL* percent excess weight loss, *BMI* body mass index, *nadir* lowest weight measured after surgery, *TW* total weight

**Fig. 2:**
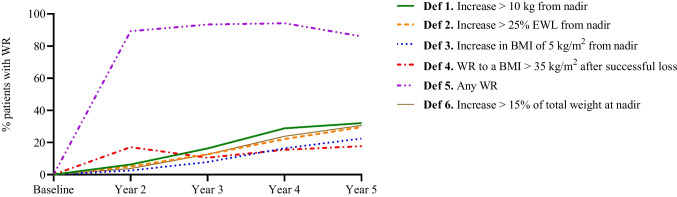
Percentage of patients with weight regain over a period of 5 years for the total population and by weight regain definition

The definition yielding the least prevalence of WR differed by year but generally, definitions 3 and 4 were responsible for the lowest WR rates across the five time points. The remaining three definitions generated WR rates that fell in between the maximum and minimum rates (Fig. 2).

### Associations of Weight Regain Definitions with Patient Characteristics at 5 Years

Table 5 shows that at 5 years, male sex was less likely to be associated with two definitions (defs 1 and 6; *P* range < 0.026 to 0.032). Patients with higher preoperative BMI had a higher likelihood of experiencing weight regain in three definitions (defs. 1, 3, and 4; *P* < 0.001–0.049). The number of comorbidities was positively associated with one definition (def. 2; *P* = 0.01). The remaining parameters under examination were not associated with any WR definitions.

**Table 5:** Associations of weight regain definitions with selected patient characteristics at 5 years

WR Definition	Demography		Preoperative			Longer term
	Age	Sex	BMI	WL	Number of comorbidities	Follow-up
Def 1 Increase of > 10 kg from nadir	1.008 (0.989, 1.026)	*0.584 *^*a*^ (0.364, 0.937)	*1.039 *^*c*^ (1.000, 1.079)	1.082 (0.522, 2.246)	1.124 (0.971, 1.301)	1.038 (0.982, 1.098)
Def 2 Increase of > 25% EWL from nadir	1.018 (0.999, 1.037)	0.654 (0.404, 1.060)	0.962 (0.923, 1.004)	0.636 (0.280, 1.446)	*1.213 *^*d*^ (1.045, 1.408)	1.031 (0.974, 1.091)
Def 3 Increase in BMI of 5 kg/m^2^ from nadir	1.011 (0.991, 1.032)	0.963 (0.562, 1.648)	*1.054 *^*d*^ (1.011, 1.098)	0.524 (0.196, 1.399)	1.126 (0.960, 1.321)	1.050 (0.990, 1.114)
Def 4 WR to BMI > 35 kg/m^2^ after successful loss	1.014 (0.992, 1.037)	1.679 (0.883, 3.192)	*1.216 *^*e*^ (1.152, 1.285)	1.362 (0.587, 3.160)	1.099 (0.923, 1.308)	1.055 (0.992, 1.123)
Def 5 Any WR	0.986 (0.963, 1.011)	0.967 (0.504, 1.855)	0.956 (0.911, 1.003)	1.494 (0.504, 4.428)	1.025 (0.835, 1.257)	0.980 (0.911, 1.054)
Def 6 Increase of > 15% of TW at nadir	1.002 (0.983, 1.021)	*0.594 *^*b*^ (0.369, 0.957)	1.014 (0.976, 1.054)	0.972 (0.461, 2.050)	1.098 (0.947, 1.273)	1.041 (0.985, 1.101)

Logistic regression analysis, cell values represent odds ratio (95% confidence interval), *Def* definition, *WR* weight regain, *Preop* preoperative, *WL* weight loss, *follow-up* number of postoperative clinic visits; *TW* total weight, italicized cells indicate statistical significance, ^*a*^*P* = 0.02, ^*b*^*P* = 0.03, ^*c*^*P* = 0.049, ^*d*^*P* = 0.01, ^*e*^*P* < 0.001

On the other hand, definition 1 was significantly associated with two characteristics (sex, preoperative BMI), while definitions 2, 3, 4, and 6 were each significantly associated with only one parameter (def. 2 with number of comorbidities; definitions 3 and 4 with preoperative BMI; def. 6 with sex).

### Associations of Weight Regain Definitions with Remission of Comorbidities at 5 Years

At five years, remission of hypertension was the only one associated with any of the WR definitions (def. 5, any WR, *P* = 0.025) (Table 6). No other WR definition was significantly associated with the remission of the comorbidities under examination.

**Table 6:** Associations of weight regain definitions with remission of comorbidities* at 5 years

WR Definition	T2DM (*n* = 89)	HTN (*n* = 105)	Dyslipidemia (*n* = 82)
Def 1. Increase of > 10 kg from nadir	1.192 (0.501, 2.838)	1.687 (0.769, 3.700)	1.552 (0.606, 3.973)
Def 2. Increase of > 25% EWL from nadir	0.980 (0.412, 2.334)	1.135 (0.520, 2.481)	1.237 (0.481, 3.182)
Def 3. Increase in BMI of 5 kg/m^2^ from nadir	1.167 (0.457, 2.978)	0.972 (0.429, 2.202)	1.154 (0.407, 3.272)
Def 4. WR to BMI > 35 kg/m^2^ after successful loss	0.911 (0.347, 2.396)	0.631 (0.251, 1.586)	0.714 (0.252, 2.023)
Def 5. Any WR	2.275 (0.548, 9.436)	*6.071 *^*a*^ (1.260, 29.246)	1.460 (0.418, 5.101)
Def 6. Increase of > 15% of TW at nadir	1.453 (0.592, 3.568)	1.461 (0.659, 3.240)	1.591 (0.594, 4.263)

^*^Analysis for patients who had comorbidities at baseline, cells represent odds ratio (95% confidence interval), *Def* definition, *WR* weight regain, *HTN* hypertension, *T2DM* type 2 diabetes, *TW* total weight, italicized cells indicate statistical significance, ^*a*^*P* = 0.025

## Discussion

To date, definitions of WR after BMS are premised on a variety of different parameters, and within each parameter, employ a wide range of different cutoffs. Despite the variability in the way WR is defined and measured, it is frequently used as a key outcome and employed in comparisons of mid- and long-term effectiveness of bariatric procedures vis-a-vis each other [7, 9, 27, 36]. Such a situation inevitably raises the question: When definitions differ, are comparisons meaningful and do differences matter? In response, the current study appraised the extent of WR among a large sample of patients and explored its dynamics across five years using 6 different definitions. We also assessed the associations between different WR definitions and  patient's characteristics as well as the remission of comorbidities at five years.

The main findings were that in terms of the time points, during years 2, 3, 4, and 5, the percentage of patients with WR fluctuated between 2.53 and 94.18%, subject to the definition and time point. At the 5-year follow-up, definition 5 (any WR) generated the highest prevalence of WR. The regression analysis at 5 years showed that sex, preoperative BMI and number of comorbidities were related to experiencing WR using various definitions. On the other hand, for remission of comorbidities, only hypertension remission was related to any of the WR definitions under examination. As expected, the definition of WR certainly influenced the WR prevalence, yielding considerable variations in the WR rates.

In terms of the *breadth* of the WR, at five years, 17.7–86% patients in the present study experienced WR subject to the definition used. Such a wide range of WR is very similar to the 16–87% WR prevalence five years after RYGB/LSG reported by Voorwinde et al. [21]. However, Lauti and colleagues [12] observed a higher WR (40–91%) using similar definitions as in the current study. Likewise, others observed WR ranging between 43.6–86.5% at five years after RYGB [14]. Two reasons might account for the lower WR rates observed by the current study and by Voorwinde et al. [21] compared to that of Lauti [12]. The first could be the lower mean preoperative BMI in our and Voorwinde’s studies [21] (43.12 and 44.8 kg/m^2^ respectively), compared to the higher baseline BMI of patients (50.7 kg/m^2^) in Lauti’s study [12]. Higher preoperative BMI is associated with a greater likelihood of WR [11, 37]. A second reason that cannot be ruled out could be the variations between centers and countries regarding the extent of multidisciplinary program before and after surgery and extent of follow-up on the recommended dietary, physical activity, and behavior modifications that might influence the sustainability of positive changes accomplished after BMS, hence playing a role in preventing WR or otherwise [11, 30, 38–41]. It is also not straight forward to rule out how patient characteristics might play a role if any, as 64.23% of our patients were females and mean age was 34 years; while these studies [12, 14, 21] had slightly higher percentage of females (79–81%) and an older population (46–49 years).

As for the *magnitude* of WR, based on the definition used, our highest proportion of patients with WR was with definition 6 (any WR, 86%), concurring with the findings of previous studies where this same definition yielded the highest WR rate (87–91%) [12, 21]. Notwithstanding, others found that the WR definition of “ ≥ 10% WR of maximum weight lost” was the one associated with highest proportion of patients with WR at five years (86.5%) [14]. On the other hand, in the current study, definition 4 yielded the lowest WR rate (17.7%), concurring with the findings of previous studies where this same definition resulted in the lowest WR rate (16%) [21].

Regarding the *dynamics* of WR across time, the proportion of patients with WR in the present study increased throughout the study period, regardless of the definition. This agrees with a study among RYGB patients and although the rate increased throughout the five years, the authors observed that the largest WR occurred during the first year after reaching nadir weight [14].

Collectively, the above findings highlight two points. The first is that some degree of WR occurs (and perhaps should be reasonably unsurprising) among most patients after BMS [11, 12, 21, 42] The second is that our findings as well as the literature illustrate a situation that requires overdue attention. They highlight the importance of debating and where possible standardizing the reporting of WR across the bariatric literature. The use of different WR definitions applied to the same population significantly alters the proportion of patients categorized as regaining weight. Such inconsistencies result in significant and clinically important findings.

As for the regression analysis, we observed that male sex was less likely to be associated with two definitions (definitions 1 and 6; *P* range < 0.026 to 0.032). Findings are inconsistent across studies, with some reporting no relation between sex and any of the 6 WR definitions [21]; or male gender being a predictor of WR [43]; or no association between gender and WR [23]. In connection with age, the current study noted no associations between age and WR across any of the definitions, where others found that age was inversely related to WR [21, 43].

As for preoperative BMI, it was positively associated with three WR definitions that incorporated weight and/or BMI (defs. 1, 3 and 4; *P* range < 0.001 to 0.049). This concurs with other studies where preoperative BMI was positively related to WR when definitions were based on changes in BMI, %EWL, and kilograms [21]. Likewise, one study showed that pre-surgery BMI was sometimes a positive significant predictor and at other times a negative predictor of WR subject to the definition used and its components (WR of “10 kg nadir,” “an increase of > 20% of maximum weight loss,” or “increase of > 25% EWL from nadir”) [23]. A recent study found that a preoperative BMI of > 45 kg/m^2^ was associated with WR after LSG, regardless of the definition of WR [44].

In terms of the number of comorbidities, we found it was positively associated with one definition (def. 2; *P* = 0.01). While there is no literature to directly compare this finding with, inconsistencies again exist. Some authors found that WR was associated with several comorbidities including obstructive sleep apnea and fatty liver disease [44], while others noted that the presence of T2DM or HTN were both not associated with WR definition [23].

Regarding to the remission of comorbidities, in the present study, WR was only significantly associated with remission of hypertension (def. 5 “any WR,” *P* = 0.025), albeit in a direction opposite to what would be expected. This suggests that comorbidity remission might not be entirely weight dependent. Such view is further supported by our observation that the remission of T2D and dyslipidemia were also not associated with any of the WR definitions. Our findings concur with other studies suggesting that WR might not largely be related to comorbidity remission. For instance, Voorwinde found that WR was associated with only one of the five comorbidities they examined (obstructive sleep apnea) [21]. Other mechanisms frequently play a role in resolution. For instance, several factors contribute to hypertension remission, including gut hormones (peptide YY and glucagon-like peptide-1), resolution of other obesity-related comorbidities that share pathophysiologic mechanisms with HTN (e.g., obstructive sleep apnea), decreases in systemic inflammation leading to reduced arterial stiffness, as well as decreased sodium reabsorption and diminished sympathetic activation [45–48].

As to the question of which WR definition is most appropriate to assess patient with WR, Table 7 provides a summary of design, patient populations, type of surgery, time points of assessment, and findings of the current and another four published studies that attempted to push the boundaries in order to answer this critical question. The table clearly illustrates the extent of the range of the variables that were assessed, when and how many times, as well as the inconsistencies in the findings and the wide variability in the reported range of values of WR and many dissimilarities in the associations/non-associations of different WR definitions with patient characteristics, resolution/remission of comorbidities, and patient perspectives (e.g., health-related QoL or satisfaction after surgery). The table undoubtedly shows that identifying one single categorical definition of clinically significant WR is difficult. Certainly, answering the question of which WR definition is the most appropriate to assess patient with WR requires further efforts and research from the bariatric community in order to reach a consensus.

**Table 7:** Towards a single definition of WR: Summary characteristics and findings of current study and comparison with the literature

Parameter	Current study Qatar	Lauti (2017) New Zealand [12]	King (2019) USA [14]	Voorwinde (2020) Netherlands [21]	Torrego-Ellacuría (2021) Spain [23]
Procedure/s	LSG	LSG	RYGB	RYGB, LSG	Multiple ^*a*^
Patients (*N*)	587	96	1406	868	445
Baseline BMI (kg/m^2^)	43.12	50	46.3	44.8	44.94
Number of definitions tested	6	6	8	6	6
Follow-up duration (years)	5	5	5	5	6 (5–8)
Number of time point/s WR was measured	4	1	5	1	1
Time at which associations of WR Defs with other variables measured	5 yrs	5 yrs	1,2,3,4,5 yrs	5 yrs	4 yrs
% Patients with WR at 5 years	17.7–86	9–91	43.6–86.5	16–87	25.4–68
Association of WR with:					
Age	✘	✘	**—**	✓ (3 Defs)	✘
Sex	✓ (2 Defs)	✘	**—**	✘	✘
Preoperarive BMI	✓ 3 (Defs)	✘	**—**	✓ (4 Defs)	✓ (4 Defs)
Race	**—**		**—**	✘	No
Bougie size	**—**	**—**	NA	**—**	**—**
T2DM	✘	**—**	✓ (8 Defs)	✘	✓ (4 Defs)
HTN	✓ (1 Def)	**—**	✓ ^*b*^	✘	✘
Dyslipidemia	✘	**—**	✓ ^*b*^	✘	**—**
OSA	**—**	**—**	**—**	✓** (**2 Defs)	**—**
Arthroses	**—**	**—**	**—**	✘	**—**
Type of surgery		NA	**—**	✓ (1 Def)	✓ (4 Defs)
Number of comorbidities	✓ (1 Def)	**—**	**—**	**—**	**—**
Follow-up^*c*^	✘	✘	**—**	**—**	**—**
Patient perspective	**—**	-BAROS a/w 3 Defs	-PHRQoL a/w 8 Defs	-HRQoL	**—**
		-Patient opinion of success	-MHRQoL a/w 3 Defs	-Physical health subscale a/w 3 Defs	
			Satisfaction with surgery a/w 8 Defs	-Mental health subscale not a/w any Def	

Due to space limitations, only the first author is cited, *Def/s* definition/s, *Preop* preoperative, **—** not reported, ✓ yes associated, ✘ no not associated, *a/w* associated with, ^*a*^includes LSG, RYGB, BPD-DS, SADI-S, ^*b*^number of definitions associated with the given comorbidity not reported by the authors, ^*c*^number of postoperative clinic visits, *WR* weight regain, *HTN* hypertension, *T2DM* type 2 diabetes, *BAROS* bariatric analysis reporting outcome system, *LSG* laparoscopic sleeve gastrectomy*, RYGB* Roux-en-Y gastric bypass, *BPD-DS* biliopancreatic diversion with duodenal switch, *SADI-S* single anastomosis duodeno-ileal bypass with sleeve gastrectomy, *PHRQoL* physical health–related quality of life, *MHRQoL* mental health–related quality of life

This study has limitations. Nadir weight could have been reached between yearly assessments time points. Appraisal of remissions of obstructive sleep apnea and osteoarthritis would have been beneficial, although their prevalence was low among our sample. We had some missing data due to loss of follow-up and we are unable to confirm whether such missing values could be related to WR, as some studies found that patients that attend their postoperative appointments have better %EWL compared to those who failed to attend [49]. Data on patient perspectives would have been useful to collect, e.g., health-related quality of life or patient satisfaction with surgery. Future research should address these limitations. Despite this, the present study has many strengths. It is one of the very few studies to test different WR definitions, and one of the fewer to appraise such definitions across patients who had undergone LSG. Unlike others [21] where subjective patient-reported medication use and blood pressure were employed, the status of comorbidities and their remission in the current study was premised on objective findings (blood tests and medication used ascertained from pharmacy dispense records).

### Final Thoughts—is Weight Regain a Useful Clinical Concept?

‘Exploring the unknown requires tolerating uncertainty’.

Brian Greene

Realistically, some WR is expected after BMS, thus multidisciplinary bariatric teams need to encourage long-term healthy lifestyle after surgery. Notwithstanding, the study findings uncover two critically challenging approaches.

On the one hand, using the findings of the current study pragmatically, awareness that higher preoperative BMI may be associated with longer term WR after LSG could prompt patients, when feasible, to seek earlier treatment, although this might not be entirely under their control. For the bariatric team, preoperative knowledge of patient groups at risk of WR, namely males with higher BMI and multiple comorbidities will help clinicians, where indicated, to offer earlier surgical intervention. Postoperatively, awareness of at-risk groups will also aid in providing early prevention or intervention when patients are not on their expected/optimal weight loss trajectory.

Conversely, on the other hand, the study findings raise concern about whether asking the question “Which WR definition is the most appropriate?” represents a suitable approach, and whether identifying a single categorical definition of clinically significant WR is a useful exercise. Perhaps the validity of WR as an outcome employed in comparisons and gauging of effectiveness of BMS procedures deserves a serious revisit. In particular, our findings as well as those of more recent studies [21] highly suggest that the current dichotomous WR definitions represent minor clinical significance as: (a) they showed mostly no associations or odd weak association with clinical outcomes; and (b) such associations were significant with only one of the comorbidities examined. Deliberations are required by expert groups, regional and international BMS agencies as to whether using metric definitions of WR after BMS represent an advantageous concept.

Given the above contrasting views, it might be argued that dichotomous WR definitions could offer some guidance in the assessment and management of individual patients. However, as a comparator metric across patients and procedures, its utility might need to be refined. Some authors have suggested a potential alternative concept that is increasingly being employed across the bariatric literature [50], where health benefits are assessed according to weight loss (e.g., sustained 5%, 10%, 15%, and 20% weight loss) [10, 51].

## Conclusion

The prevalence WR after BMS remains inconsistent and its clinical significance uncertain. Our findings indicate that some WR could be reasonably expected after BMS. If preventing WR per se is viewed as an important dimension of success of BMS due to amelioration of comorbidities, then our findings indicate that the present dichotomous WR definitions are of minor clinical significance due to the weak associations with limited comorbidities. A deeper debate is required, as identifying a single categorical definition of clinically significant WR is difficult, and its full potential remains to be determined. Future research should address this challenge.
